# Identifying aggressive prostate cancer foci using a DNA methylation classifier

**DOI:** 10.1186/s13059-016-1129-3

**Published:** 2017-01-12

**Authors:** Kamilla Mundbjerg, Sameer Chopra, Mehrdad Alemozaffar, Christopher Duymich, Ranjani Lakshminarasimhan, Peter W. Nichols, Manju Aron, Kimberly D. Siegmund, Osamu Ukimura, Monish Aron, ‬Mariana Stern, Parkash Gill, John D. Carpten, Torben F. Ørntoft, Karina D. Sørensen, Daniel J. Weisenberger, Peter A. Jones, Vinay Duddalwar, Inderbir Gill, Gangning Liang

**Affiliations:** 1 USC Institute of Urology and the Catherine & Joseph Aresty Department of Urology, Norris Comprehensive Cancer Center, University of Southern California, Los Angeles, CA 90089 USA; 2 Department of Pathology, Norris Comprehensive Cancer Center, University of Southern California, Los Angeles, CA 90089 USA; 3 Department of Preventive Medicine, Norris Comprehensive Cancer Center, University of Southern California, Los Angeles, CA 90089 USA; 4 Department of Medicine, Norris Comprehensive Cancer Center, University of Southern California, Los Angeles, CA 90089 USA; 5 Department of Translational Genomics, Norris Comprehensive Cancer Center, University of Southern California, Los Angeles, CA 90089 USA; 6 Department of Biochemistry and Molecular Biology, Norris Comprehensive Cancer Center, University of Southern California, Los Angeles, CA 90089 USA; 7 Department of Molecular Medicine, Aarhus University Hospital, 8200 Aarhus N, Denmark; 8 Van Andel Research Institute, Grand Rapids, MI 49503 USA; 9 Department of Radiology, Norris Comprehensive Cancer Center, University of Southern California, Los Angeles, CA 90089 USA

**Keywords:** DNA methylation, Prostate cancer, Aggressiveness, Multifocal

## Abstract

**Background:**

Slow-growing prostate cancer (PC) can be aggressive in a subset of cases. Therefore, prognostic tools to guide clinical decision-making and avoid overtreatment of indolent PC and undertreatment of aggressive disease are urgently needed. PC has a propensity to be multifocal with several different cancerous foci per gland.

**Results:**

Here, we have taken advantage of the multifocal propensity of PC and categorized aggressiveness of individual PC foci based on DNA methylation patterns in primary PC foci and matched lymph node metastases. In a set of 14 patients, we demonstrate that over half of the cases have multiple epigenetically distinct subclones and determine the primary subclone from which the metastatic lesion(s) originated. Furthermore, we develop an aggressiveness classifier consisting of 25 DNA methylation probes to determine aggressive and non-aggressive subclones. Upon validation of the classifier in an independent cohort, the predicted aggressive tumors are significantly associated with the presence of lymph node metastases and invasive tumor stages.

**Conclusions:**

Overall, this study provides molecular-based support for determining PC aggressiveness with the potential to impact clinical decision-making, such as targeted biopsy approaches for early diagnosis and active surveillance, in addition to focal therapy.

**Electronic supplementary material:**

The online version of this article (doi:10.1186/s13059-016-1129-3) contains supplementary material, which is available to authorized users.

## Background

Prostate cancer (PC) is the most frequently diagnosed non-skin cancer and the second most common cause of cancer deaths in men in the United States. Although PC incidence rates have increased over the past 25 years, mortality rates have largely remained unchanged (https://www.cancer.gov/). The development of prostate specific antigen (PSA) testing as a screening tool for PC has resulted in increased diagnoses of PC; however, many of these are less aggressive lesions with unclear clinical significance. Thus, a central dilemma in the management of clinically localized PC is whether to postpone treatment and monitor until the disease becomes more aggressive in order to minimize patient health side effects, or to treat immediately to avoid progression and dissemination of disease. Treatment of localized PC with radical prostatectomy or radiation therapy is associated with high cure rates; however, this is associated with significant side effects, including urinary incontinence (5–20%), erectile dysfunction (30–70%), and bowel toxicity (5–10%) [1, 2]. Generally, PC is a slow-growing malignancy with decades of indolence, but the aggressive forms display rapid growth, dissemination, and lethality in a subset of cases (<20%) [3, 4]. Furthermore, no curative therapies are available for metastatic PC patients. This highlights the need for novel prognostic tools to guide clinical decision-making and avoid both overtreatment of indolent PC and undertreatment of aggressive disease [4].

Predicting tumor aggressiveness and likelihood of progression is critical for clinical decision-making. PC is graded using the Gleason system, in which tumors with higher Gleason Scores (GSs) tend to be more aggressive [5, 6]. GS is calculated by summing the primary (largest pattern) and secondary (second largest pattern) Gleason grades, each of which ranges from 1 (well differentiated) to 5 (poorly differentiated) [5]. However, the relationship between individual GSs of clinically localized PCs and those that progress to metastatic disease is poorly understood [7]. The tumorigenic events during PC progression have been difficult to investigate, and the ability to characterize late stages of PC progression is lacking due to limited availability of metastatic tissues. In addition, 60–90% of PCs are multifocal [8], in which one prostate contains several seemingly unconnected locations of cancer growth. The development of multifocal PC is still highly debated and two models have been described [8]. One theorizes that an initially transformed cancer spreads to multiple locations within the prostate (monoclonal), while the other model suggests that PC foci arise independently in different areas of the same gland (multiple subclones) [9–18]. The latter option indicates the possibility that aggressive and non-aggressive cancer foci co-exist in the same prostate gland and is supported by the finding that individual foci of multifocal PC often present with unique GSs [19]. Consequently, the index lesion (the cancer lesion with the largest volume or the highest GS depending on the study) may not be representative of PC behavior [20] and subsequently complicates sample selection for analysis and clinical decision-making. Therefore, previous studies that have not accounted for prostate tumor multifocality, or used only the index lesion, are potentially flawed.

Recently, focal therapy has been put forth as a novel approach for destruction of only the index lesion (highest GS) in localized unifocal and multifocal PCs in order to reduce adverse health side effects. GSs of individual PC lesions, including index lesions, can differ amongst multifocal PC lesions [19], and treatment decisions are usually based on the assumption that the index tumor drives PC progression [21]. Therefore, accurate characterization of the index tumor or aggressive lesion is a fundamental issue for PC management.

DNA methylation alterations occur in every cancer type and, importantly, DNA methylation levels change concordantly with tumor aggressiveness in most types of cancer [22]. Epigenetic alterations can drive tumorigenesis and determine tumor aggressiveness and, therefore, can be used for diagnostic purposes [23] as well as to inform therapeutic approaches [24, 25]. Although PC has been shown to harbor a great hereditary element [26, 27], only an estimated 30% of these factors have presently been accounted for in PC patients [28]. Interestingly, recent studies have been able to connect genetic alterations and DNA methylation changes, indicating that DNA methylation changes hold information regarding the clonal evolution of PC. For example, multiple metastases within a PC patient have been shown to arise from a single precursor cancer cell, or focus, by copy number alterations (CNAs), mutation and gene expression patterns, and DNA methylation changes [21, 29, 30], suggesting that only one focus of a multifocal PC is responsible for the development of the metastatic lesions. Moreover, unified evolution of DNA methylation and CNAs was identified in five cases of monofocal PC and their matched lymph node metastases [11].

In this study, we have approached the issue of PC aggressiveness from a novel perspective. We have taken advantage of the multifocal propensity of PC and categorized aggressiveness of individual PC foci based on DNA methylation patterns in primary PC foci and matched metastases. In a set of 14 patients with multifocal PC, we demonstrate that over half of the multifocal PC cases have multiple subclones and determine the primary subclone from where the metastatic lesion(s) originated. Overall, we describe a unique approach to identify aggressive PC lesions using DNA methylation markers, which have potential utility in clinical decision-making regarding whether the patient should undergo treatment or be monitored by active surveillance.

## Results

### DNA methylation patterns of lymph node metastases indicate the potential primary focus/foci of origin

**Fig. 1:**
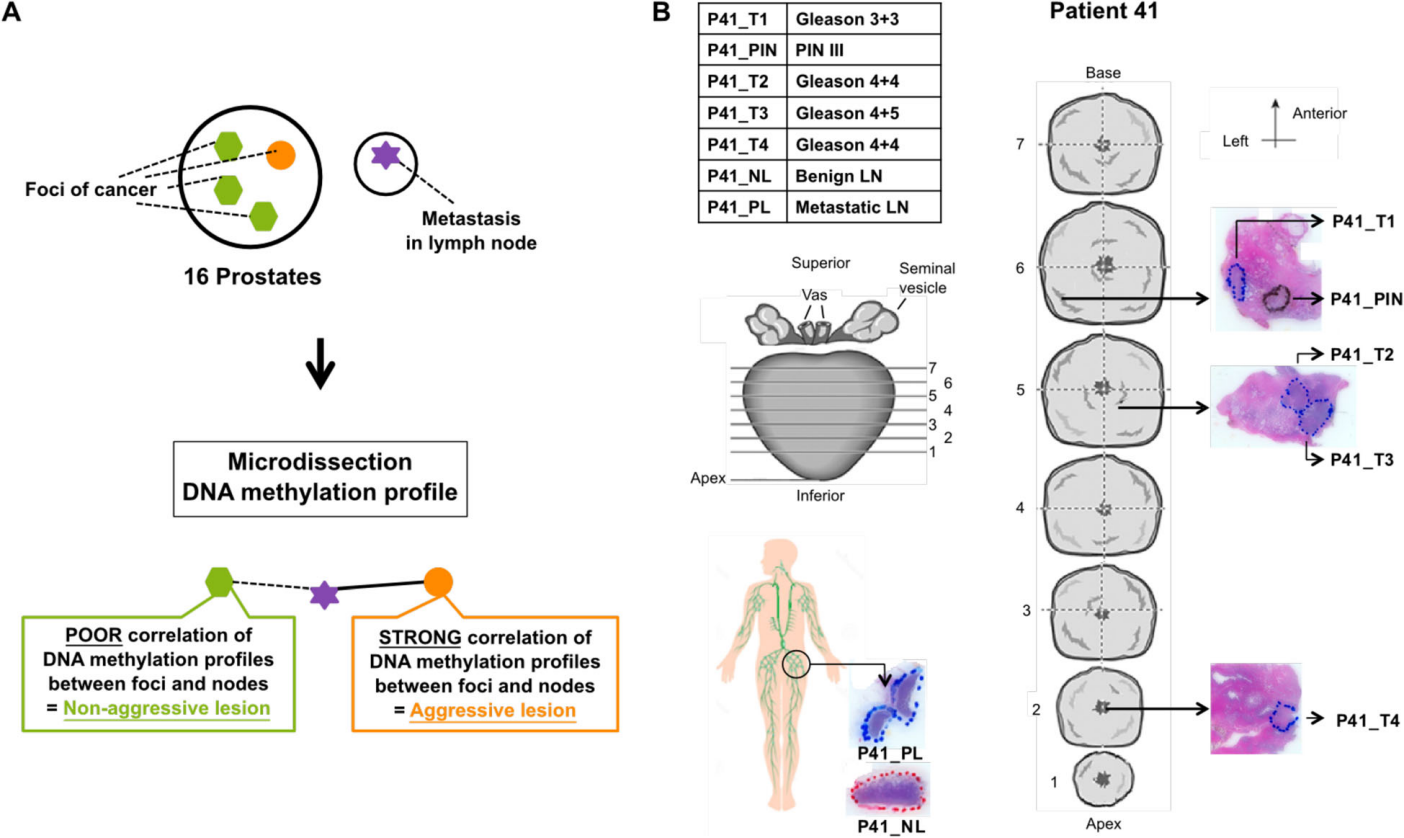
Strategy and sample selection. **a** A prostate gland with four cancer foci (*green* and *orange* areas) and a pelvic lymph node with metastasis marked by a *purple star*. Our hypothesis is that we can determine the primary focus of metastasis origin based on matching DNA methylation in the lymph node metastasis, and this in turn will represent the most aggressive cancer subclone. By determining the aggressive subclone in multifocal PCs, we will obtain groups of aggressive and non-aggressive samples, which will form the basis for developing a classifier to determine the aggressiveness of primary PC foci. **b** An overview of the samples from patient 41 is shown in the upper left corner. *P* patient, *T* primary tumor focus, *NL* tumor-negative lymph node, *PL* tumor-positive lymph node. The physical location of the five prostate samples and the two lymph node samples collected are shown on schematics of the dissected prostate gland (*middle*) and the lymphatic system (*lower left corner*), respectively

In this study, we hypothesize that the aggressive primary cancer focus/foci can be identified from multifocal PC by the degree of correlation of DNA methylation to lymph node metastases, which are representative of an aggressive trait (Fig. 1a). Our hypothesis relies on four assumptions: 1) a subset of multifocal PCs arise from independent and sporadic genetic/epigenetic changes, effectively implying that distinct cancer foci develop through different molecular mechanisms/pathways and harbor unique proliferative, migration, and aggressiveness potential; 2) DNA methylation changes inform about clonal evolution and will not change substantially upon dissemination [11, 30, 31]; 3) PC metastases have the same clonal origin [21, 30]; and 4) pelvic lymph nodes drain from a cancerous prostate and are likely the initial site of metastatic spread. Thus, nodal metastases, along with advanced pathologic stage, constitute aggressive traits, which are surrogates for metastatic potential.

We used the Illumina Infinium HumanMethylation450 BeadArray (HM450) platform to measure genome-scale DNA methylation of matched primary tumors and pelvic lymph node metastases in 16 patients who underwent radical prostatectomy for multifocal disease (Additional file 1: Table S1). Prostate and nodal tissue samples stored in formalin-fixed, paraffin embedded (FFPE) tissue blocks were sectioned, stained with hematoxylin and eosin (H&E) (Fig. 1b), and examined by two specialized genitourinary pathologists. All areas of cancer were marked and assigned a GS, including primary tumor foci (T), adjacent-normal (AN) prostate tissues, tumor-negative lymph nodes (NLs), tumor-positive lymph nodes (PLs), and, when possible, prostatic intraepithelial neoplasia (PIN), summing to a total of 92 samples (“Methods”). Sample purity was tested for either infiltration of normal cells or leukocytes caused by inflammation using DNA methylation data (“Methods”; Additional file 1: Figure S1). Two primary tumor foci were removed due to low tumor cell content (P17_T3 and P23_T3) and two PL metastases were removed due to high leukocyte content (P15_PL and P32_PL), thereby excluding all samples from patients 15 and 32. HM450 DNA methylation data from the remaining 14 patients were compared in a multidimensional scaling (MDS) plot, in which samples are placed in two-dimensional space based on dissimilarity (Additional file 1: Figure S2). Primary tumors and lymph node metastases were highly heterogeneous with no obvious subgroups, whereas normal prostate and lymph node tissues formed a tight cluster, as expected, indicating that cancer-specific DNA methylation alterations are evident in our sample cohort.

**Fig. 2:**
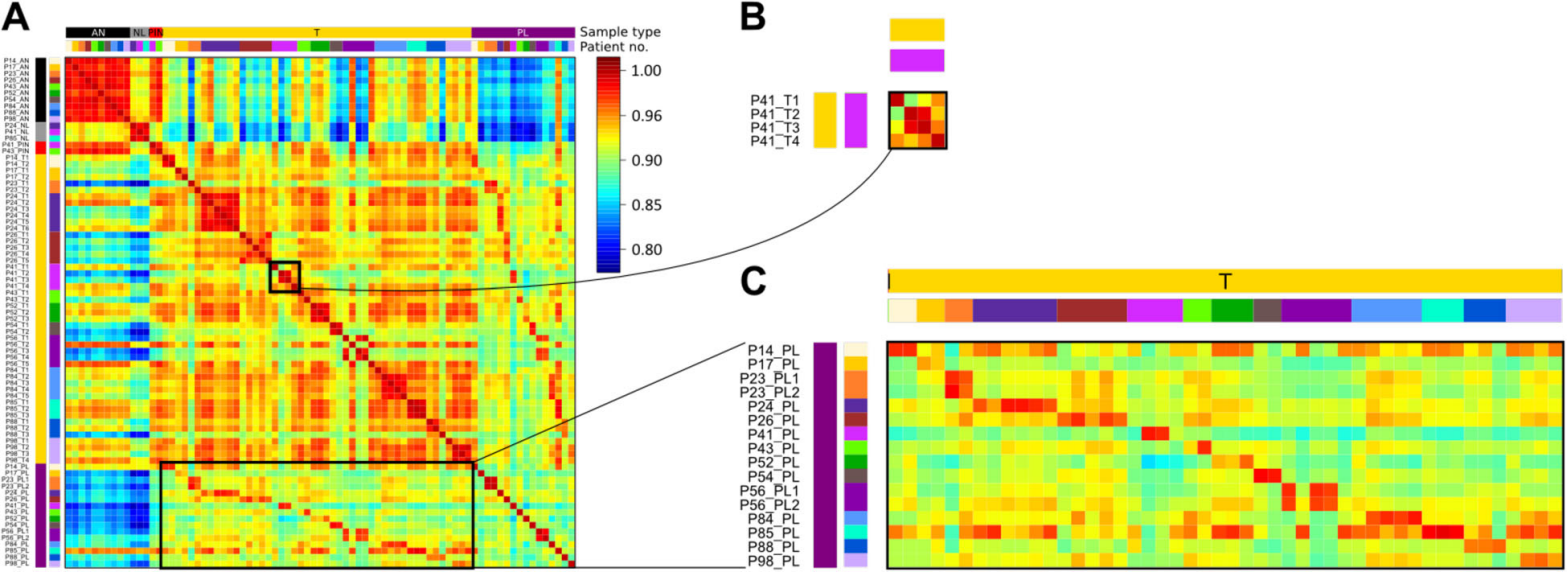
DNA methylation of metastasis and primary site from the same patient is highly similar. **a** Between-sample correlation plot. Sample names are shown to the *left* of the plot. At the *top* and the *left* of the plot are *colored sidebars* showing sample type and patient identifier. The *sidebar* to the *right* of the plot shows the correlation coefficient color key, *red* being high correlation and *blue* low correlation. *P* patient, *AN* adjacent normal, *T* primary tumor focus, *NL* tumor-negative lymph node, *PL* tumor-positive lymph node. **b** Enlargement of correlation amongst primary tumor foci in patient 41. **c** Enlargement of correlation between all primary tumor foci and all positive lymph nodes

In order to investigate if DNA methylation patterns hold information about clonal evolution in PC, Pearson correlations amongst all the samples were calculated, plotted, and visualized using heatmaps (Fig. 2a). Firstly, primary foci from the same patient showed more variable correlation coefficients (0.89–0.99) compared to interpatient AN–AN samples (0.96–0.99) and interpatient AN–NL samples (0.90–0.94), indicating that multiple cancer subclones are present in some patients (Fig. 2b) and in turn may hold distinct tumorigenic potential. Secondly, lymph node metastases consistently showed the highest correlation to one or more of the primary tumor foci from the same patient (0.94–0.98; Fig. 2c). Thus, DNA methylation profiles had not diverged to such a degree that metastases and primary tumors remained comparable. Taken together, these results demonstrate that a subset of multifocal PCs show independent epigenetic changes, indicating that cancer foci develop from unique subclones. Furthermore, the DNA methylation profiles of lymph node metastases are highly correlative to a focus/foci from individual patients.

**Fig. 3:**
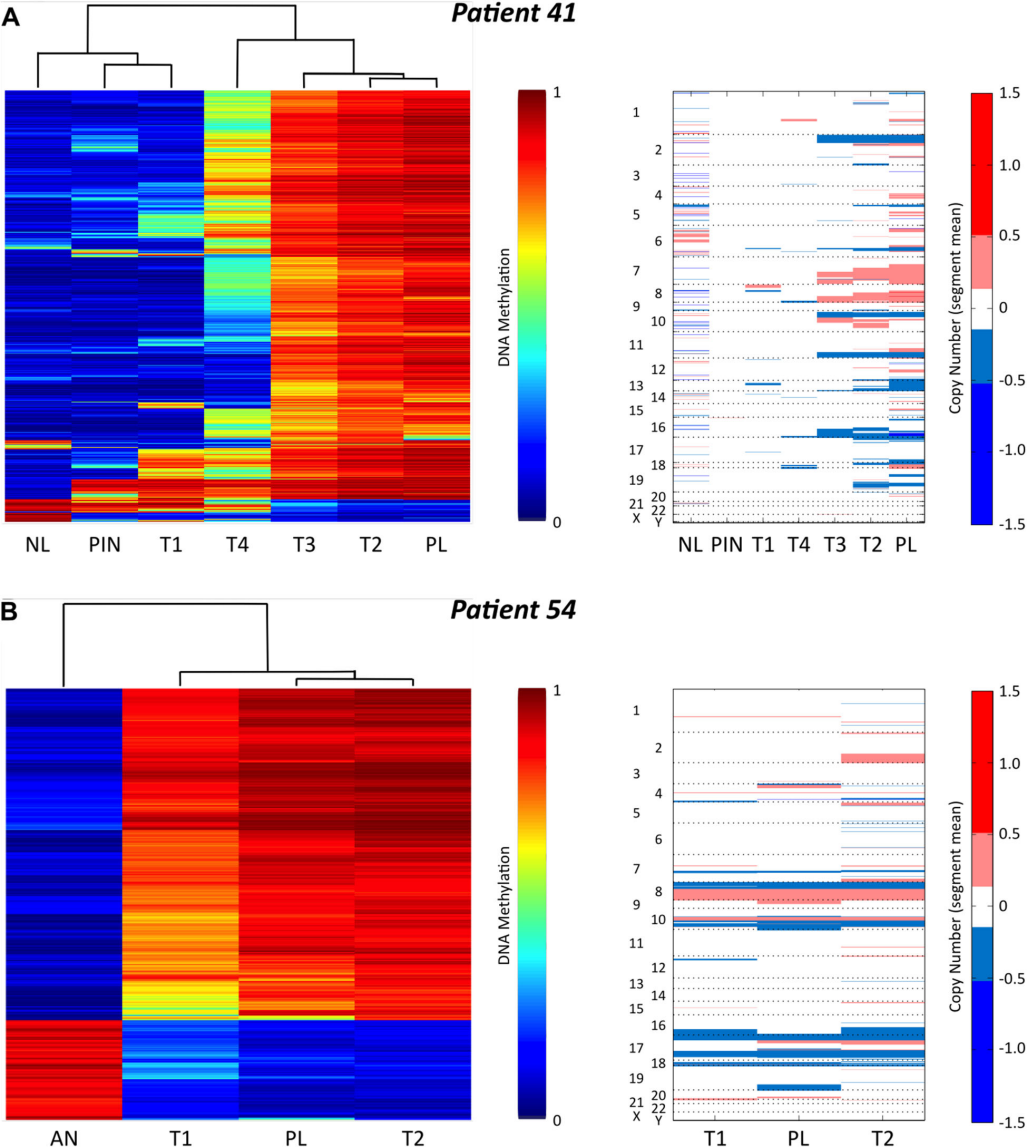
DNA methylation patterns of lymph node metastasis indicate the potential primary focus/foci of origin. *Left*: Unsupervised clustering and heatmaps of all the samples from patient 41 (**a**) and patient 54 (**b**) based on the top 1% most variably methylated probes between all samples except the PLs. Dendrograms are shown above the heatmaps and the *color key* is to the *right. Right*: Copy number alterations in patient 41 (**a**) and patient 54 (**b**). In each plot, samples are ordered based on the unsupervised clustering from the heatmaps to the *left*. The *numbers* and *letters* on the *left* of the plot designate the chromosome numbers. To the *right* is shown the *color key*: *red* = chromosomal gain and *blue* = chromosomal loss

Next, we investigated the DNA methylation profiles of PC foci among individual patients. To identify the focus of origin of lymph node metastasis, we selected the top 1% most variably methylated probes between all samples, excluding PLs, for each patient. The DNA methylation levels of these probes from all samples, including PLs, were then compared by unsupervised hierarchical clustering and heatmap visualization. Based on similar DNA methylation levels, we expect PLs to cluster with one or more primary tumors, thereby providing information regarding the potential clonal relationship between primary PCs and PLs. Heatmaps after unsupervised clustering of these probes for two representative patients, patients 41 and 54 (Fig. 3a, b, left panels), as well as for the remaining 12 patients with lymph node metastases (Additional file 1: Figure S3) are shown. In all 14 cases with lymph node metastases, the PLs clustered with one or more of the matched primary tumor foci and no PLs clustered with the AN prostate tissues, normal lymph nodes, or PIN lesions (Fig. 3; Additional file 1: Figure S3). In addition, PLs clustered and were highly correlated in two patients (P23 and P56) with multiple PLs (0.99 and 0.98, respectively; Additional file 1: Figure S3), supporting the assumption (assumption 3) that metastases have the same clonal origin.

The PL DNA methylation profile for patient 41 clustered very closely with the T2 and T3 primary tumor foci, while the T4 and T1 foci were more dissimilar, as shown by the dendrogram at the top of the heatmap (Fig. 3a). For this patient, the T2 and/or T3 foci are the most likely origin(s) of the metastasis. Furthermore, the physical juxtaposition of T2 and T3 within the prostate specimen (Fig. 1b) suggests these two foci diverged from the same population of transformed cells during tumorigenesis. In addition, patient 41 also displayed tumor foci with very different DNA methylation profiles, indicating the occurrence of multiple independent transformation events and, therefore, multiple subclones (Fig. 3a). Patient 54 had two primary foci (T1 and T2) and the PL DNA methylation data were very similar to both tumor foci. Hence, both patients displayed multiple primary tumor foci with very similar DNA methylation profiles, indicating a monoclonal origin of these PCs.

In order to validate these findings, we took advantage of the recent evidence that the HM450 DNA methylation platform can also be used to determine CNAs by summing the methylated and unmethylated signal intensities of the probes [32, 33]. This analysis provided additional evidence that the T2 and T3 foci were very similar to the PL in patient 41. Both T2 and T3 foci had deletions on chromosomes 2, 10, 11, and 16 and gains on chromosomes 7, 8, and 10; however, these regions were not altered in the T1 or T4 foci, which show different CNA patterns (Fig. 3a, right panel). All three samples from patient 54 presented with multiple shared alterations, as well as deletion of the short arm and amplification of the long arm of chromosome 8, both common features of PC [34, 35] (Fig. 3b, right panel). Overall, the CNA analysis supports our findings of multiple subclonal origins in patient 41 (Fig. 3a) and a monoclonal origin in patient 54 (Fig. 3b) based on DNA methylation analysis. Moreover, the CNA results also support our finding that the origin of lymph node metastasis can be determined by DNA methylation data.

Similarly, all PLs clustered with one or more primary tumor foci from the remaining 12 cases using our DNA methylation-based approach (Additional file 1: Figure S3). Furthermore, nine patients (P23, P24, P26, P41, P43, P56, P84, P88, and P98) showed clearly distinct DNA methylation patterns among the primary foci, indicating the existence of independent tumor subclones. Taken together, these results suggest that the PL DNA methylation pattern can be used to identify the potential primary focus/foci of origin of metastasis and that PC patients may contain subclones with aggressive and non-aggressive potential.

### Development of a panel of DNA methylation markers as a classifier for PC aggressiveness

**Fig. 4:**
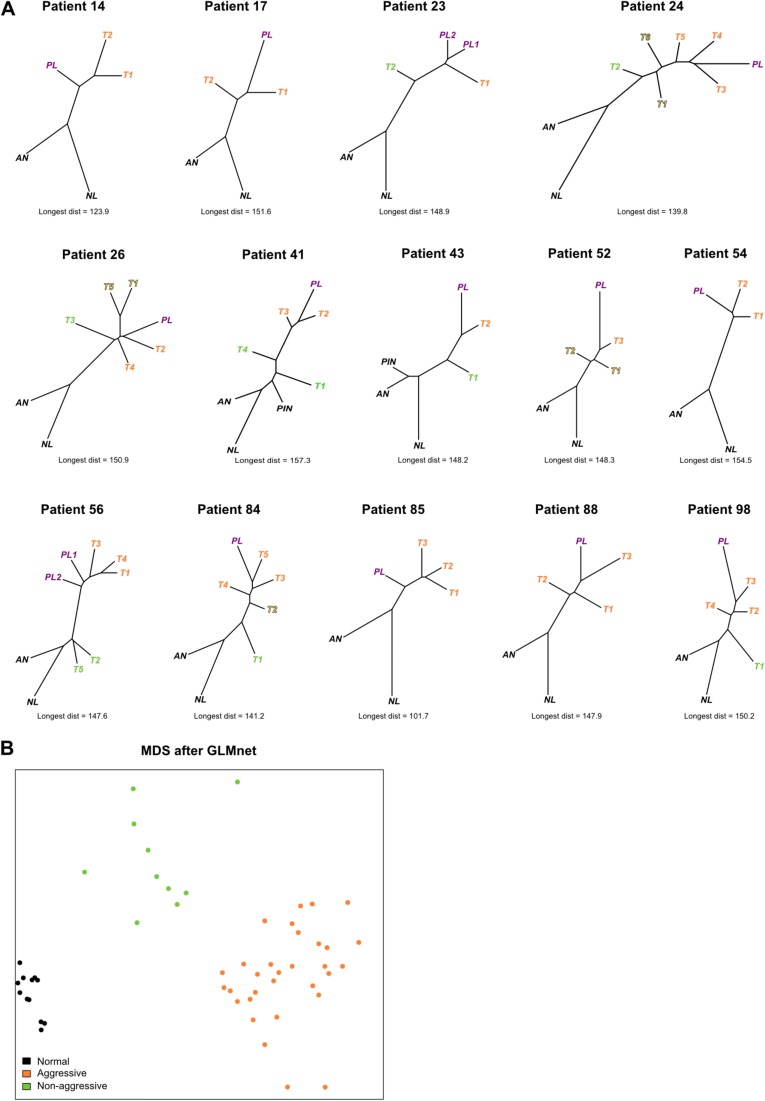
Building an aggressiveness classifier. **a** Phylogenetic reconstruction showing clonal relationships in each patient based on all filtered HM450 probes. Averaged normal prostate and normal lymph node samples were used for each tree. Sample types are colored with *black* (normal and PIN), *orange* (aggressive primary tumor), *green* (non-aggressive primary tumor), *yellow shaded* (undecided primary tumor), and *purple* (lymph node metastasis). Below each tree the longest Euclidean distance between any two samples in the tree are denoted so as to serve as a reference between the different trees. **b** MDS plot based on a 25-probe classifier generated by GLMnet of the samples used for the analysis. The samples are separated into three distinct groups and show no overlap

Next, we devised a DNA methylation-based PC aggressiveness classifier to categorize primary PC foci as either aggressive or non-aggressive. The unsupervised hierarchical clustering approach effectively identifies the primary origin of lymph node metastases; however, in order to categorize the aggressiveness of individual foci in a quantitative, unbiased, and objective manner, we calculated Euclidean distances between any two samples within a patient using all filtered HM450 probes. Euclidean distance, like Pearson correlation, compares sample similarities, but maintains data variability, and is also superior for analysis of differential gene expression analysis [36]. We divided the scale of Euclidean distances into discrete categories (aggressive, non-aggressive, and undecided) for all primary tumor foci. Since the purpose of this categorization method is to assemble groups of genuinely aggressive and non-aggressive tumors for biomarker development, we included a gap of 10 Euclidean distance units (undecided category) to reduce the risk of misclassification. Sample categorization for each patient is shown using DNA methylation-based phylogenetic trees, where samples are colored as a function of aggressiveness (Fig. 4a; overview in Additional file 1: Table S2).

Taken together, our developed categorization approach found that eight patients (patients 23, 24, 26, 41, 43, 56, 84, and 98) showed independent DNA methylation profiles indicative of multiple subclones. Five patients (patients 14, 17, 54, 85, and 88) showed similar DNA methylation patterns, indicating a monoclonal origin, and one patient (patient 52) was categorized as undecided (Fig. 4a; Additional file 1: Table S2). These findings are in agreement with the unsupervised clustering data (Fig. 3; Additional file 1: Figure S3) with the exception of patient 88, who did not show discrete subclones as indicated by the heatmap and dendrogram. In this patient, the top 1% most variably methylated probes were not representative of the potential clonal relationship.

We next searched for differentially methylated probes between the aggressive and non-aggressive groups (false discovery rate (FDR)-adjusted *p* < 0.05) but found that the DNA methylation levels of no single probe were significantly different between the two groups. Using an FDR cutoff of 0.3, 231 probes were identified. Still, we continued to search for a set or panel of probes able to distinguish these groups from a larger panel. First, we generated a list of the 3000 most differentially methylated probes between the assembled aggressive and non-aggressive groups based on mean DNA methylation differences (Additional file 1: Figure S4), which was subsequently used as input for the GLMnet algorithm [37] along with information about normal, aggressive, and non-aggressive sample groups. The GLMnet model generates outputs in the form of probabilities of group membership, which are functions of the DNA methylation values for a given set of probes that differentiate the groups. Upon numerous iterations and refinement of the input probes list (“Methods”), we found a set of 25 probes (Additional file 1: Table S3) that optimally predict normal, non-aggressive, and aggressive categories (Fig. 4b). Of the 25 probes in the classifier, 21 (84%) were among the probes with FDR-adjusted *p* < 0.3 for either aggressive versus non-aggressive, aggressive versus normal, or non-aggressive versus normal comparisons.

### The Cancer Genome Atlas PC cohort validates the potential of our aggressiveness classifier

**Fig. 5:**
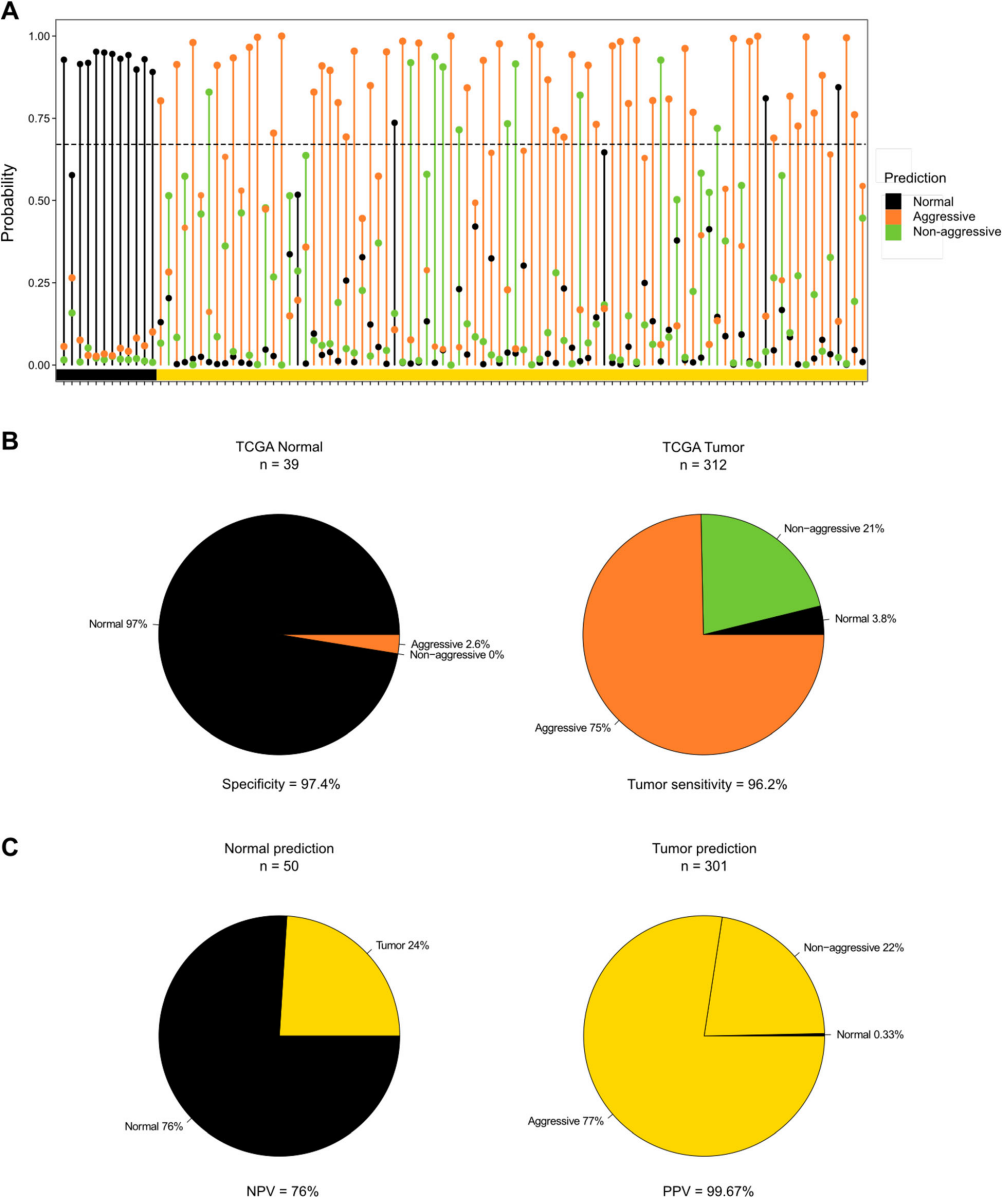
Validation of the PC aggressiveness classifier**. a** Manhattan plot of the probabilities calculated for 100 randomly selected samples from TCGA PC cohort. The *color bar* at the *bottom* of the plot designates the sample types determined by TCGA. *Black* = adjacent normal prostate, *yellow* = primary PC. The *black dotted line* marks the probability threshold used. **b** Distribution of the prediction of TCGA tumor and AN samples. **c** Evaluation of correctly predicted samples based on the histological microscopic examinations performed by TCGA. *NPV* negative predictive value, *PPV* positive predictive value

To test the classifier on an independent dataset, we took advantage of the publically available prostate adenocarcinoma (PRAD) HM450 DNA methylation data and accompanying clinical information from The Cancer Genome Atlas (TCGA) project. We tested 496 prostate samples (tumor and AN) using the classifier. For each sample, the probabilities of normal, aggressive, and non-aggressive groups sum to 1, and the group with the highest probability is the predicted phenotype of a given sample. Of the TCGA PRAD samples (*n* = 351; 312 tumors and 39 AN samples), 70% were predicted with a probability above 0.67 (see 100 random samples as an example in Fig. 5a). Of the 39 AN prostate TCGA samples, 38 were predicted as normal and one as aggressive. Of the 312 primary tumors (see Additional file 1: Figure S5 for distribution of clinical information), 233 were predicted as aggressive, 67 were predicted as non-aggressive, and 12 were predicted as normal, thus resulting in a 97.4% specificity and a 96.2% cancer sensitivity for PCs compared to AN tissue samples (Fig. 5b). Upon evaluation of the consistency between our predictions and the sample diagnoses (PC versus AN) based on the histological microscopic examinations performed by TCGA, the classifier has a 76% negative predictive value and a 99.7% positive predictive value (Fig. 5c). The preponderance of high GSs (about 50% of tumors in G8–10; Additional file 1: Figure S5a) and advanced T3–T4 stage (over 70% of tumors; Additional file 1: Figure S5b) in TCGA PRAD tumor may explain the high proportion of cancers predicted as aggressive (Fig. 5). Indeed, we do find this result strengthens the validity of our classifier.

**Fig. 6:**
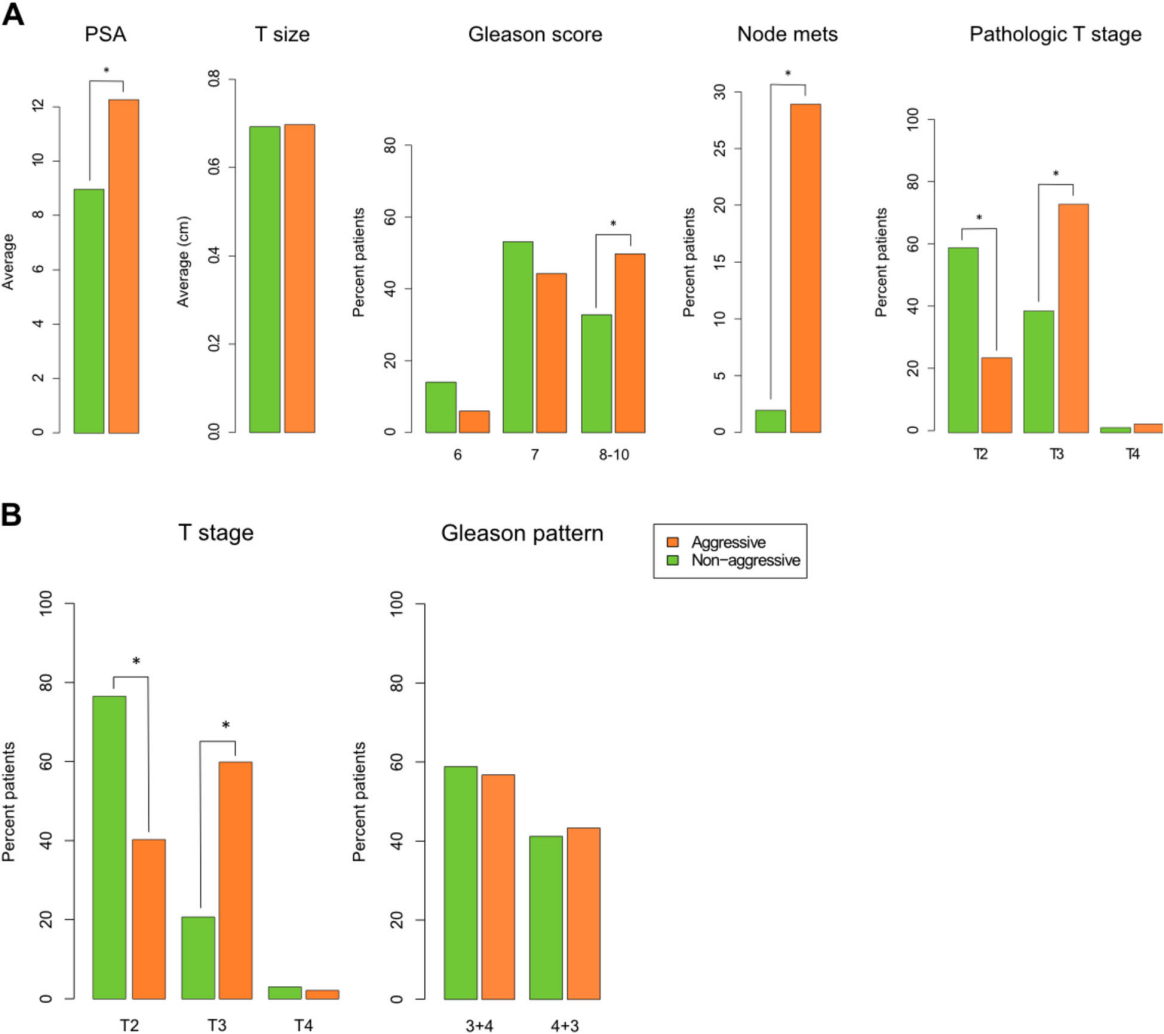
Clinical information for predicted TCGA groups. **a** Pre-operative PSA among the aggressive (n = 215) and non-aggressive (n = 64) groups. Welch two sample t-test = 0.005. Tumor size represented by the average intermediate dimension in centimeters among the aggressive (n = 87) and non-aggressive (n = 25) groups. Welch two sample t-test = 0.9428. Percentage of patients with lymph node metastases at the time of surgery among the aggressive (n = 187) and non-aggressive (n = 52) groups. Fisher’s exact two-tailed *p*(Yes) = 9.2 × 10^−5^. Pathological T stage distribution among the aggressive (n = 217) and non-aggressive (n = 64) groups. Fisher’s exact two-tailed *p*: *p*(T2) = 2.2 × 10^−7^, *p*(T3) = 7.7 × 10^−7^, *p*(T4) = 0.6969. GS distribution among the aggressive (n = 217) and non-aggressive (n = 64) groups. Fisher’s exact two-tailed *p*: *p*(GS 6) = 0.0591, *p*(GS 7) = 0.2539, *p*(GS 8–10) = 0.0220. *P* values <0.05 are marked by an *asterisk*. **b** Distribution of GS 3 + 4 and 4 + 3 tumors among the aggressive (n = 96) and non-aggressive (n = 34) groups. Fisher’s exact two-tailed *p*(3 + 4) = 0.8424. *P* values <0.05 are marked by an *asterisk*. Pathological T stage of GS 7 tumors among the aggressive (n = 96) and non-aggressive (n = 34) groups. Fisher’s exact two-tailed *p*: *p*(T2) = 1.5 × 10^−4^, *p*(T3) = 1.2 × 10^−4^, *p*(T4) = 1

To evaluate the prognostic performance of the classifier, we consulted available clinicopathological covariates associated with PC aggressiveness, including pre-operative PSA, tumor size, pathological GS, presence of lymph node metastases, and tumor stage, for samples with probabilities above 0.67. Aggressiveness was significantly (*p* < 0.02) associated with the investigated covariates except tumor size (Fig. 6; Additional file 1: Figure S6). Pre-operative PSA levels were higher in the aggressive group compared to the non-aggressive group (*p* = 0.005; Fig. 6a; Additional file 1: Figure S6). However, similar tumor sizes between groups (Fig. 6a; Additional file 1: Figure S6) indicate that aggressiveness and tumor size are independent as has also been suggested previously [13]. Interestingly, we found a significant association between PC aggressiveness and GS using a Chi square test (*p* = 0.018). Importantly, we found that significantly more patients classified as having an aggressive PC presented with lymph node metastases at the time of surgery compared to patients with predicted non-aggressive tumors (*p* = 9.2 × 10^−5^; Fig. 6a). Also, the pathological evaluation of tumor stage (Fig. 6a) showed significantly more organ-confined stage T2 tumors in the non-aggressive group (*p* = 2.2 × 10^−7^) and significantly more of the capsule-penetrating and seminal vesicle invasive stage T3 tumors in the aggressive group (*p* = 7.7 × 10^−7^).

Upon further examination, tumors with high GSs (GS 8–10) were significantly associated with the aggressive group (*p* = 0.022), but no such association was seen for tumors with low (GS 6) and intermediate (GS 7) scores (*p* = 0.059 and *p* = 0.254, respectively; Fig. 6a). GSs are well correlated with PC aggressiveness, especially at the low (GS 6) and high (GS 8–10) ends of the scale [5, 6], and Gleason scoring is a valuable tool in PC treatment. However, additional information is required to determine aggressiveness for the intermediate (GS 7) tumors. Interestingly, the GS 7 tumors, which comprise nearly one-half of all TCGA PC tumors (Additional file 1: Figure S5), were not significantly associated with non-aggressive or aggressive groups (Fig. 6a), indicating that this large group in particular may benefit from our DNA methylation-based classifier in order to determine whether active surveillance or ablative treatment is the best course of action. In support of this, we also found that the GS 7 tumors classified as non-aggressive were significantly associated with tumor stage T2 (P = 1.5 × 10^−4^), while GS 7 tumors classified as aggressive were significantly associated with tumor stage T3 (*p* = 1.2 × 10^−4^; Fig. 6b). Furthermore, we tested whether the primary and secondary patterns of the GS 7 tumors showed a correlation to the aggressive or non-aggressive groups (Fig. 6b). GS is calculated by summing the primary (largest pattern) and secondary (second largest pattern) Gleason grades, each of which ranges from 1 (well differentiated) to 5 (poorly differentiated) [5]. Interestingly, there was no difference in the distribution between 3 + 4 and 4 + 3 tumors and indicates that tumors of this large intermediate Gleason 7 group can be further and more accurately stratified using our molecular-based classifier to help determine whether active surveillance or ablative treatment should be performed.

Taken together, the strong correlation between cancer aggressiveness and tumor stage holds great promise for our classifier if developed into a molecular DNA methylation-based assay for needle biopsy samples, since the pathological tumor stage cannot be obtained until after surgery.

## Discussion

Identification of PC aggressiveness is fundamental to improving clinical decision-making in patients diagnosed with organ-confined PC regarding treatment or active surveillance. By implementing our study design of examining DNA methylation in primary multifocal PC and matched lymph node metastases, we were able to examine the relationships amongst primary foci as well as the relationships between primary foci and metastases. Importantly, we found that more than half of the patients in our cohort showed multiple subclones, findings similar to previously reported studies [9, 11–14, 16–18], and also that DNA methylation of a lymph node metastasis is similar to a cancerous focus/foci from the same patient. Taking advantage of these findings, we developed a method to categorize the subclonal relationship and aggressiveness of individual PC foci. The resulting aggressive and non-aggressive sample groups, along with adjacent-normal samples, were used to search for biomarkers to distinguish the three groups, and the outcome was a 25-probe aggressiveness classifier. The classifier showed promising prognostic potential when it was applied to samples from the PC cohort from TCGA and merits validation in future studies including longitudinal monitoring of patients.

For this study, we relied on the assumption that DNA methylation can inform on clonal evolution. Several studies have addressed the connection between DNA methylation and clonal evolution with high precision [11, 21, 30] and, recently, Costello and colleagues reported that phyloepigenetic relationships robustly recapitulate phylogenetic patterns in gliomas and their recurrences [31]. Two or more foci originated from the same subclone in 11 of 14 patients in our cohort (Fig. 4a), indicating that an initial subclone seeded multiple locations through migration. We cannot definitively rule out that these are not actually one large or branched focus, since a fine physical connection can be hard to clearly distinguish in a pathological sample. Therefore, we do not attempt to determine which focus from the same subclone gave rise to the PL.

While clinical tools and techniques have improved immensely [1, 3, 38–41], the determination of tumor aggressiveness prior to physical manifestation must rely on biomarkers measured biochemically or at a molecular level. One impediment to success is how to define tumor aggressiveness with respect to a clinical end point. Often GS or time to PSA recurrence is used as a surrogate for PC aggressiveness, which would be more appropriately evaluated using metastatic progression or mortality. In this study, we used a novel approach in defining aggressiveness as the ability to give rise to lymph node metastases. The presence of lymph node metastases is an indication of tumor cells having acquired the ability to leave the primary site and proliferate in a secondary site and thus acts as an indicator for the capacity of the cancer to establish distant metastases. In addition to this type of lymphatic dissemination, metastases can also arise through hematogenous dissemination to brain, lungs, liver, and bone marrow [42]. Secondary cancer growths at these sites are not routinely removed during treatment for metastatic PC and, thus, the tissue for research is not available until postmortem. Although we recognize that distant metastases do not exclusively arise through lymphatic dissemination, we show that this clinical end point is very relevant alone or in concert with other clinicopathological parameters (Figs. 5 and 6).

Gleason score 7 (GS 7) tumors are among the most difficult and poorly established backgrounds for making clinical decisions [43, 44]; however, our study demonstrated that aggressiveness of PCs with GS 7 using our classifier is highly correlated with pathological tumor stage but not specific for primary or secondary Gleason patterns (4 + 3 or 3 + 4; Fig. 6b). Because of this, our classifier may challenge the current standard for clinical care and may result in placing select PC patients into active surveillance and avoidance of unnecessary invasive treatments.

A limitation to the presented study is that our discovery set is effectively only 14 patients, from whom we have 79 total samples. A larger discovery set would improve the study and would probably result in an enlargement of the classifier to more than 25 probes due to the vast PC heterogeneity [45]. Despite the modest size of the discovery set, we were able to validate the aggressiveness classifier and, thus, our study approach using publicly available TCGA PRAD DNA methylation data from 496 primary tissues. Upon correlating our predictions with the TCGA clinicopathological information, we found a significant association (*p* < 0.02) between aggressiveness and pre-operative PSA levels, pathological GS, presence of lymph node metastases, and tumor stage; interestingly, however, we did not find any correlation with tumor size. We do recognize that different clinical endpoints would be better suited to describe poor clinical outcome, however, but regret that the average follow-up period of the TCGA PRAD cohort was only 3.16 years. As a result, we found that too few patients had recurred and thus only found a significant difference between the groups for tumor status (Additional file 1: Figure S7). Taken together, the presented data suggest the novelty of using DNA methylation data to identify aggressive lesions more specifically than any currently used approach, and is especially promising due to its potential clinical applications for early detection in PC biopsy specimens.

Upon suspicion of PC, prostate biopsies are performed as the standard-of-care method for PC diagnosis [46]. Currently, prostate needle biopsies are most commonly performed trans-rectally in a systematic, yet random format. This systematic, random biopsy strategy has a high rate of misdiagnosis, since the non-targeted needles may either miss the clinically significant cancer focus, capture only a clinically insignificant cancer focus, or completely miss all cancer foci [20, 47]. Thus, the significant sampling error of traditional systematic, random prostate biopsies renders them unreliable for accurate characterization of index tumor location, volume, and GS [47]. The recently developed image-guided targeted prostate biopsy technique, which fuses magnetic resonance and three-dimensional transrectal ultrasound images, can reliably identify the location and the primary Gleason pattern of index lesions [40, 41]. By combining image-guided targeted biopsies and our DNA methylation classifier (following further clinical validation), we expect to enhance the ability to identify aggressive foci and subsequently characterize biopsy-detected PC foci more accurately. The ability to determine aggressiveness in a biopsy sample mapped to a particular prostate location also holds great promise for making more informed clinical decisions regarding the choice between active surveillance of non-aggressive PC foci and surgery or targeted focal ablation therapy of the aggressive PC foci, although it should be noted that several steps remain before approval for clinical use. Initially, the aggressiveness classifier should be developed into a more cost- and labor-efficient test in the form of a custom DNA methylation array or multiplexed PCR-based assay (MSP or MethyLight) [48, 49]. Moreover, the test should undergo extensive clinical validation in retrospectively collected samples—prostate biopsies, blood, or urine samples—before finally being tested in a clinical trial environment.

## Conclusions

Our study demonstrates the relevance for translational medicine in spanning from collected PC samples and large-scale datasets to a DNA methylation biomarker panel with potential clinical applicability.

## Methods

### Study design

Sixteen patients diagnosed with multifocal PC having metastasized to one or more pelvic lymph nodes were enrolled in the study following informed consent (Additional file 1: Table S1). All patients had radical prostatectomies and removal of pelvic lymph nodes in the period 1991–2013. No anti-androgen treatments were administered prior to surgery. The prostate and lymph node tissue samples were stored in FFPE tissue blocks. FFPE blocks were sectioned and H&E stained (Fig. 1b). Two trained pathologists examined all slides covering the entire prostate and dissected lymph nodes, and all areas of cancer were marked and given a GS. In addition, AN and PIN regions were marked when possible, summing to a total of 92 samples. The marked H&E slides were used to guide the dissection of AN/PIN/PC cells from 8–10 unstained slides (5–10 μm).

### Deparaffinization and purification

The dissected tissue samples were deparaffinized using a double xylene wash followed by a double ethanol wash and drying of the pellets. For DNA extraction, the pellets were resuspended in 240 μl of PKD buffer and Proteinase K (Qiagen, miRNeasy FFPE kit), then incubated at 55 °C overnight and finally 85 °C for 15 min. After cooling the samples, 500 μl RBC buffer was added and the samples were run through gDNA Eliminator columns (RNeasy plus mini kit) using RPE buffer to wash and EB buffer for elution.

### DNA methylation profiling

Genomic DNA (200–500 ng) from each FFPE sample was treated with sodium bisulfite and recovered using the Zymo EZ DNA methylation kit (Zymo Research) according to the manufacturer’s specifications and eluted in a 10 μl volume. An aliquot (1 μl) was removed for MethyLight-based quality control testing of bisulfite conversion completeness and the amount of bisulfite converted DNA available for the Illumina Infinium HM450 DNA methylation assay [48]. All samples that passed the quality control tests were then repaired using the Illumina Restoration solution as described by the manufacturer. Each sample was then processed using the Infinium DNA methylation assay data production pipeline as described in [50].

After the chemistry steps, BeadArrays were scanned and the raw signal intensities were extracted from the *.IDAT files using the R package *methylumi*. The intensities were corrected for background fluorescence and red-green dye-bias [51]. The beta values were calculated as (M/(M + U)), in which M and U refer to the (pre-processed) mean methylated and unmethylated probe signal intensities, respectively. Measurements in which the fluorescent intensity was not statistically significantly above background signal (detection *p* value >0.05) were removed from the data set. In addition, probes that overlap with known SNPs as well as repetitive elements were masked prior to data analyses. Specifically, all HM450 probes that overlapped with common SNPs with a minor allele frequency of greater than 1% (UCSC criteria) at the targeted CpG site, as well as probes with SNPs (minor allele frequency >1%) within 10 bp of the targeted CpG site were masked. HM450 probes that were within 15 bases of the CpG lying entirely within a repeat region were also masked prior to data analyses. The end result was a dataset of corrected beta-values for 396,020 probes spanning ~21,000 genes.

### Calculation of tumor purity

To investigate the degree of leukocyte infiltration in each sample, public HM450 data from 96 male peripheral blood samples (GSE53740 and GSE51388) were downloaded using Marmal-aid [52]. All HM450 probes with beta values >0.2 in male peripheral blood were excluded. The remaining probes were used to subset 500 probes that were hypermethylated in 43 TCGA AN prostate samples, and thus hypomethylated in peripheral blood. Tissues of prostate origin from our study with mean DNA methylation of these probes below 0.6 were excluded from further analysis. Two lymph node metastases were excluded due to high blood content. Four *GSTP1* HM450 probes (cg06928838, cg09038676, cg22224704, cg26250609) were used for tumor purity analysis as described in Brocks et al. [11]. Primary tumors with mean DNA methylation beta values <0.4 were excluded from further analysis. Two tumor samples were excluded due to high normal content.

### Unsupervised hierarchical clustering

For each patient, probes with masked beta values (detection *p* value >0.05) were excluded and the top 1% most variably methylated probes between all the samples except the PL(s) were selected. Heatmaps were used to display the DNA methylation levels and the unsupervised hierarchical clustering was performed with the *hclust* function in R (method = “complete”).

### CNA analysis

CNAs were analyzed using the *Champ* package for R [53] using 28 AN prostate samples purified from FFPE tissues (12 from this study and 16 from unpublished data) as a reference. Imported beta values were run through *champ.norm* and *champ.CNA* (filterXY = FALSE, batchCorrect = T, freqThreshold = 0.3). The generated segment mean-files were intersected with the Infinium probe locations using BedTools and the resulting chromosomal loss and gain were illustrated in heatmaps using Matlab. Most of the samples showed noisy profiles, likely due to DNA breakage accumulated during the storage in FFPE, and the analysis could not be completed for all samples.

### PC tumor aggressiveness categorization

Euclidean distances were calculated between any two samples using all 396,020 filtered probes. Averaged normal prostate and normal lymph node samples showed minimal variance and were used for the analysis. Normal prostate samples were considered to be very similar because only 0.65% (2561/396,020) of standard deviations for all the probes were >0.15. Normal lymph node samples were considered to be very similar because only 0.98% (3875/396,020) of standard deviations for all probes were >0.15. The primary focus with the shortest Euclidean distance to the lymph node metastasis (T-PL dist 1) was categorized as aggressive. The additional distance to the other primary foci (T-PL dist 2; actual T-PL dist – T-PL dist 1 = T-PL dist 2) were assessed in a density graph and a division of the scale based hereon (Additional file 1: Figure S8). If T-PL dist 2 values were only 0–10 units longer, they were also categorized as aggressive. This ensured that the foci of monoclonal origin would all be grouped as aggressive. Next, T-PL dist 2 values longer by >20 units were categorized as non-aggressive origins and T-PL dist 2 values of between 10–20 were categorized as undecided (overview in Additional file 1: Table S2). In the two patients with two PLs the division of the primary tumors was done based on the PL with the shortest distance to a primary focus, namely P23_PL2 and P56_PL1.

### Phylogenetic reconstruction

DNA methylation-based phylogenetic trees were inferred by the minimal evolution method [54]. Euclidean distances were calculated using all 396,020 filtered probes.

### Calculation of differential methylation

Differential methylation between any two groups of samples was calculated using the *champ.MVP()* function from the ChAMP package utilizing either FDR <0.05 or FDR <0.3.

### Developing the DNA methylation-based PC aggressiveness classifier

By combining the categorized samples into groups of aggressive (*n* = 31) and non-aggressive (*n* = 10), we generated a list of 3000 most variably methylated CpG sites (probes) between the groups as follows. The mean beta values of all filtered probes were calculated for aggressive and non-aggressive groups. The differences between the two groups were calculated, the absolute values were ordered, and the top 3000 probes were used for further analysis. This list was used as input for the GLMnet algorithm [37] to predict a multinomial outcome: normal (*n* = 12), non-aggressive (*n* = 10), and aggressive (*n* = 31) prostate sample groups. The GLMnet algorithm outputs a set of probes able to differentiate groups of samples based on their DNA methylation profile. Following 15 iterations, each output was evaluated by 1) the separation of the three groups (input as normal, aggressive, non-aggressive) in multidimensional scaling (MDS) plots like those in Fig. 4b and Additional file 1: Figure S2; 2) the DNA methylation levels of the probes in heatmaps; 3) the prediction probabilities in Manhattan plots like in Fig. 5a for each set of probes run back on the input samples. Different random starts of the algorithm resulted in different final models. Following 15 random starts, a total of 39 probes were utilized by at least one model. More random starts did not provide additional probes over and above the 39. We re-ran the GLMnet algorithm utilizing these 39 probes as input and a set of 25 probes (Additional file 1: Table S3) was found to be the optimal predictor of our sample set according to normal, non-aggressive, and aggressive categories.

### Testing the aggressiveness classifier on TCGA DNA methylation data

TCGA PRAD HM450 DNA methylation data were downloaded from TCGA Data Portal (https://tcga-data.nci.nih.gov/tcga/). After filtering samples based on the same criteria as for our own samples, 499 samples (45 normal, 453 tumor, and one metastatic) remained. After removing samples with missing values among the 25 predictor probes, 496 samples remained (45 normal, 450 tumor, and one metastatic). The classifier was run on these samples and 70% were predicted with a probability above a cutoff of 0.67. A cutoff of 0.67 was chosen because as a consequence the probability for either of the two other groups must be 0.33 or less. Clinicopathological data were available for most samples in Biotab-files and are shown for the samples predicted above the 0.67 cutoff.

### Statistics

In Fig. 6a, b, Welch two sample t-tests were used to calculate statistical significance. In Fig. 6c–e, Fisher’s exact two-tailed tests were used to calculate significance. *P* values <0.05 were considered significant.

## Additional file

Additional file 1: Figure S1. Sample purity. **Figure S2.** Sample dissimilarity. **Figure S3.** Unsupervised clustering and heatmaps. **Figure S4.** Input probes for GLMnet analysis. **Figure S5.** Distribution of clinical information for the 312 TCGA tumor samples predicted by our classifier. **Figure S6.** Beeswarm plots. **Figure S7.** Clinical follow-up information for predicted TCGA tumor samples. **Figure S8.** Density graph of T-PL dist 2. **Table S1.** Patient information. *LN* lymph node. **Table S2.** PC foci aggressiveness by patient. **Table S3.** 25-probe aggressiveness classifier. (PDF 1585 kb)

## Abbreviations

AN: Adjacent-normal
CNA: Copy number alteration
FDR: False discovery rate
FFPE: Formalin-fixed, paraffin embedded
GS: Gleason Score
H&E: Hematoxylin and eosin
MDS: Multidimensional scaling
NL: Tumor-negative lymph node
PC: Prostate cancer
PIN: Prostatic intraepithelial neoplasia
PL: Tumor-positive lymph node
PRAD: Prostate adenocarcinoma
PSA: Prostate specific antigen
TCGA: The Cancer Genome Atlas.
