# Supplementary material for: Identifying aggressive prostate cancer foci using a DNA methylation classifier
Source: Genome Biol. 2017 Jan 12;18:3. doi: 10.1186/s13059-016-1129-3 (PMC5234101; doi:10.1186/s13059-016-1129-3)
Supplement: Additional file 1: Figure S1. — Sample purity. Figure S2. Sample dissimilarity. Figure S3. Unsupervised clustering and heatmaps. Figure S4. Input probes for GLMnet analysis. Figure S5. Distribution of clinical information for the 312 TCGA tumor samples predicted by our classifier. Figure S6. Beeswarm plots. Figure S7. Clinical follow-up information for predicted TCGA tumor samples. Figure S8. Density graph of T-PL dist 2. Table S1. Patient information. LN lymph node. Table S2. PC foci aggressiveness by patient. Table S3. 25-probe aggressiveness classifier. (PDF 1585 kb) [file 13059_2016_1129_MOESM1_ESM.pdf]

**Supplemental Figure legends**

**Figure S1. Sample purity.** *Top:* Boxplot of 500 probes methylated in prostate tissue and unmethylated in peripheral blood. The normal lymph node samples had median DNA methylation beta values of ~0.2. Any of the other samples with medians below 0.6 were excluded from further analysis: P15_PL and P32_PL.Tumor purity. *Bottom*: Boxplot of *GSTP1* promoter DNA methylation for all samples. Tumor samples with median DNA methylation below 0.4 (dotted vertical line) were excluded from analysis: P17_T3 and P23_T3. The two PIN lesions were both just below 0.4 and were kept for further analysis.

**Figure S2. Sample dissimilarity.** MDS plot drawn from Euclidean distances calculated based on all filtered probes.

**Figure S3. Unsupervised clustering and heatmaps.** Clustering and heatmap visualization for all samples from Patients 14, 17, 23, 24, 26, 43, 52, 56, 84, 85, 88, and 98 was based on the top 1% most variably methylated probes between all samples, except the PLs. Dendrograms are shown above the heatmaps and the color key to the right.

**Figure S4. Input probes for GLMnet analysis.** Heatmap of the top 3,000 most differentially methylated probes between the aggressive (n = 31) and non-aggressive (n = 10) groups based on the average methylation differences. Sidebar on the top shows the group of each sample (same as in Figure 4).

**Figure S5. Distribution of clinical information for the 312 TCGA tumor samples predicted by our classifier.** Gleason score distribution. GS 6 (n = 23),

GS 7 (n = 137), GS 8-10 (n = 132), unknown (n = 20). T stage distribution. T2 (n = 99), T3 (n = 202), T4 (n = 8), unknown (n = 3).

**Figure S6. Beeswarm plots.** Distribution of intermediate dimension of TCGA tumors among aggressive (n = 87) and non-aggressive (n = 25) groups. Welch two sample t-test = 0.9428. Pre-operative PSA among the aggressive (n = 215) and non-aggressive (n = 64) groups. Welch two sample t-test = 0.005. Red bars mark the mean.

**Figure S7. Clinical follow-up information for predicted TCGA tumor samples.** Available clinical follow-up data at the time of writing this manuscript for predicted groups shown in figures and contingency tables. Average follow-up period is 3.16 years. New tumor events among aggressive (n = 149) and non-aggressive (n = 44) groups. Fisher's exact 2-tailed P(Yes) = 0.2263. Vital status among the aggressive (n = 233) and non-aggressive (n = 67) groups. Fisher's exact 2-tailed P(Dead) = 1. Biochemical recurrence among the aggressive (n = 196) and non-aggressive (n = 56) groups. Fisher's exact 2-tailed P(Yes) = 0.0591. Clinical recurrence among the aggressive (n = 186) and non-aggressive (n = 57) groups. Fisher's exact 2-tailed P(Yes) = 0.0401.

**Figure S8. Density graph of T-PL dist 2.** T-PL dist 2 were calculated for all primary tumor samples (n = 48) and plotted. A distance of 0 indicates the tumor foci most like PLs.

# Figure S1

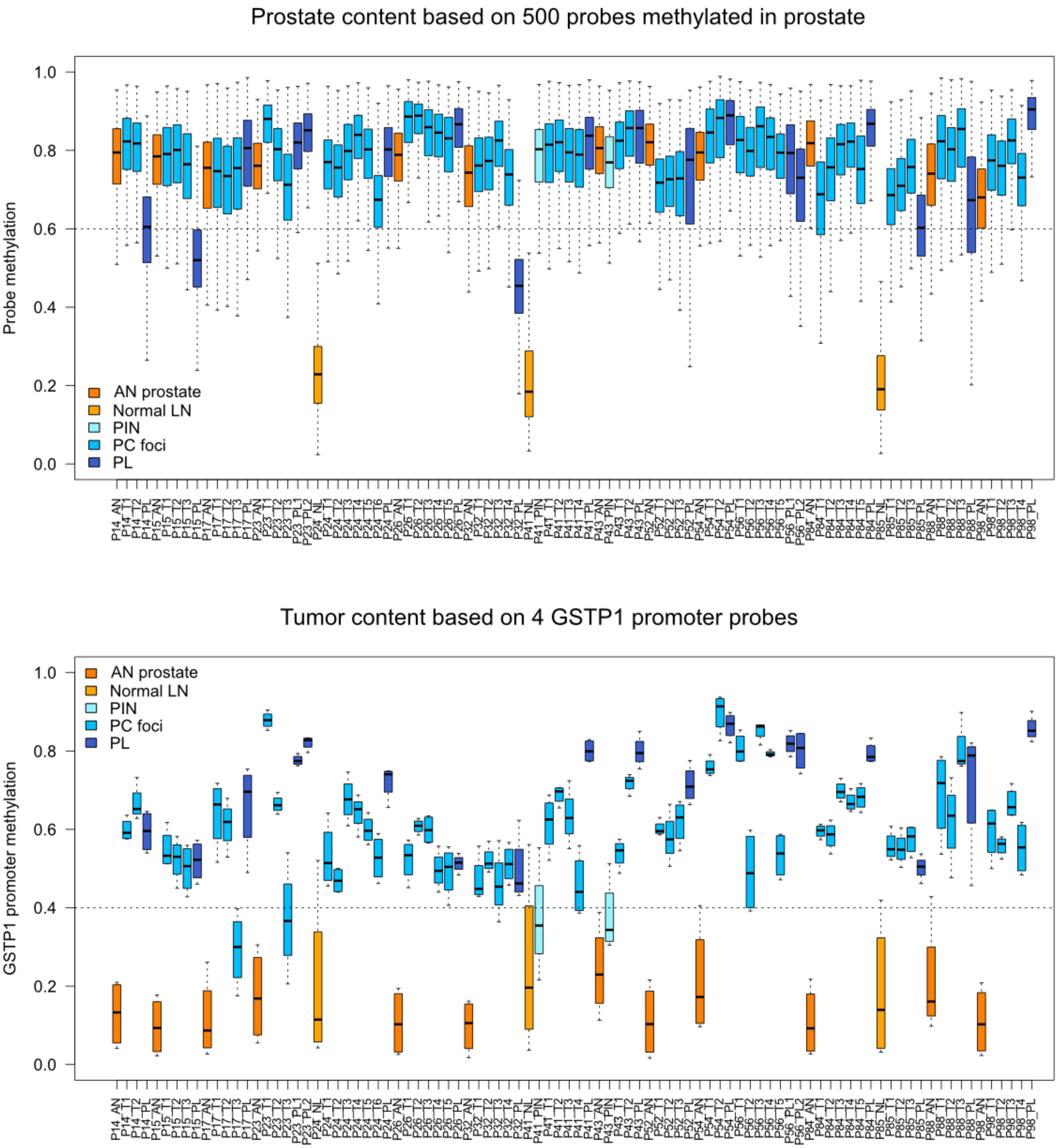
Prostate content based on 500 probes methylated in prostate
Probe methylation
AN prostate
Normal LN
PIN
PC foci
PL
Tumor content based on 4 GSTP1 promoter probes
GSTP1 promoter methylation
AN prostate
Normal LN
PIN
PC foci
PL

# Figure S2

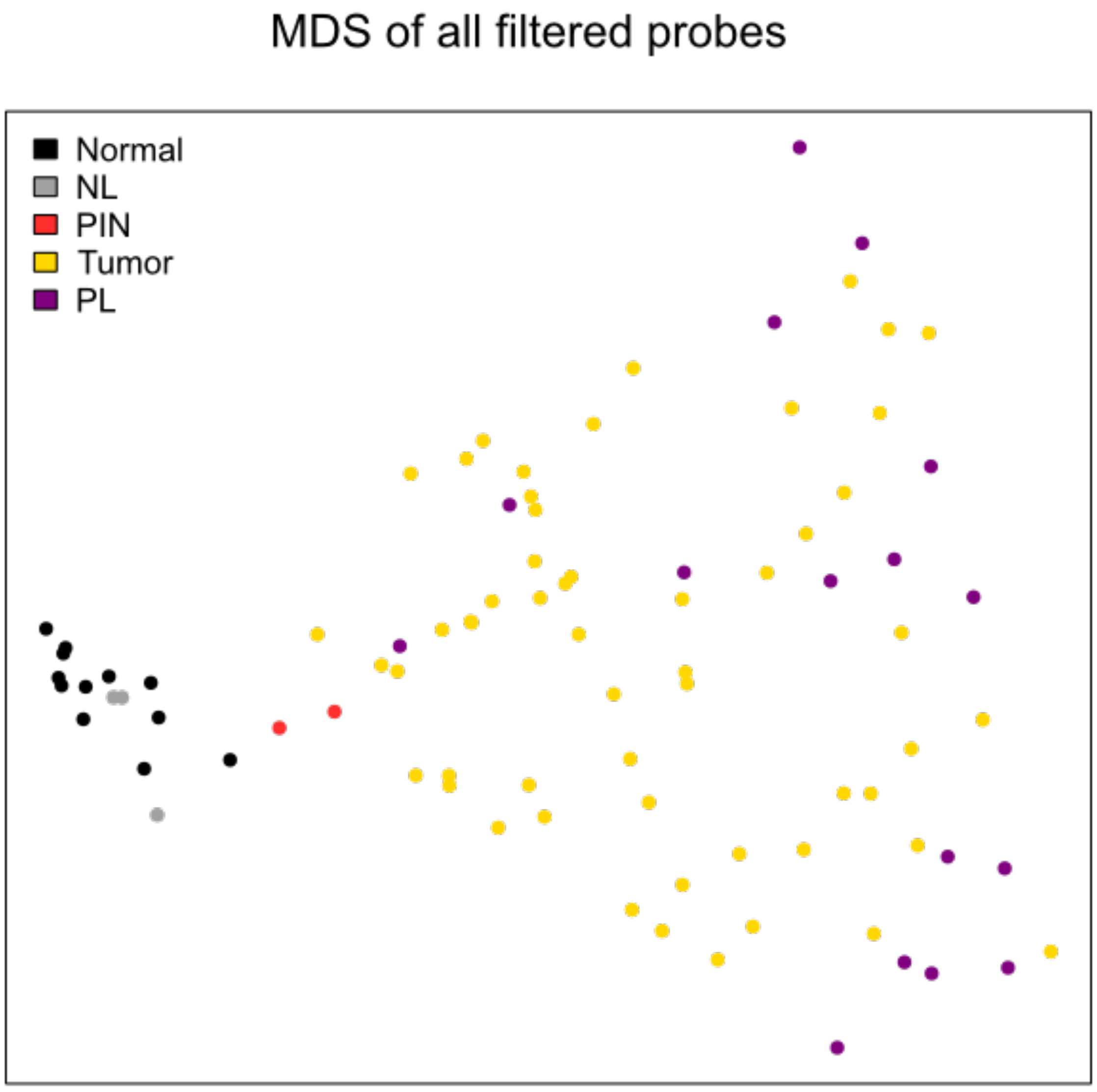

# Figure S3

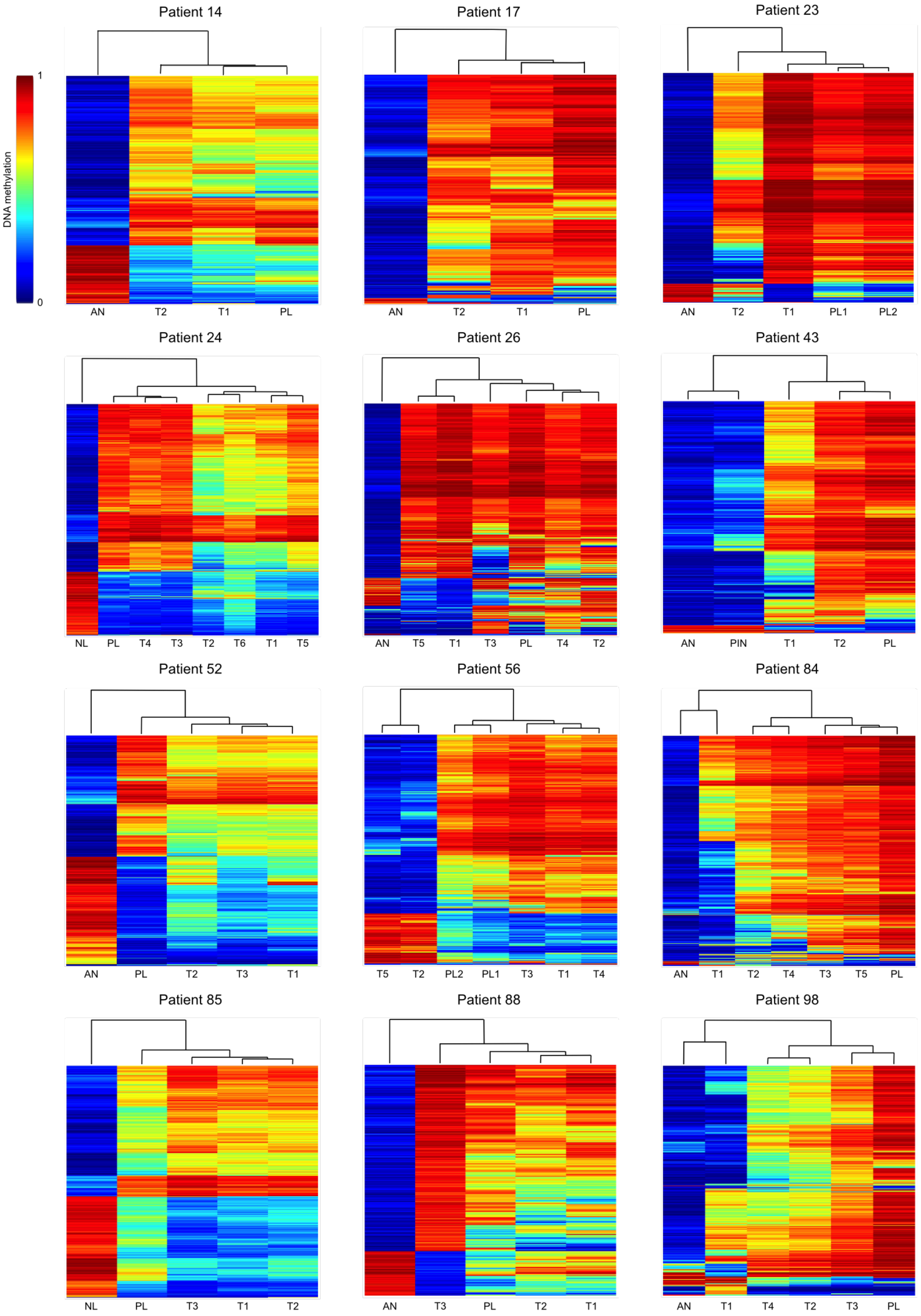
DNA methylation
1
0
Patient 14
AN T2 T1 PL
Patient 17
AN T2 T1 PL
Patient 23
AN T2 T1 PL1 PL2
Patient 24
NL PL T4 T3 T2 T6 T1 T5
Patient 26
AN T5 T1 T3 PL T4 T2
Patient 43
AN PIN T1 T2 PL
Patient 52
AN PL T2 T3 T1
Patient 56
T5 T2 PL2 PL1 T3 T1 T4
Patient 84
AN T1 T2 T4 T3 T5 PL
Patient 85
NL PL T3 T1 T2
Patient 88
AN T3 PL T2 T1
Patient 98
AN T1 T4 T2 T3 PL

# Figure S4

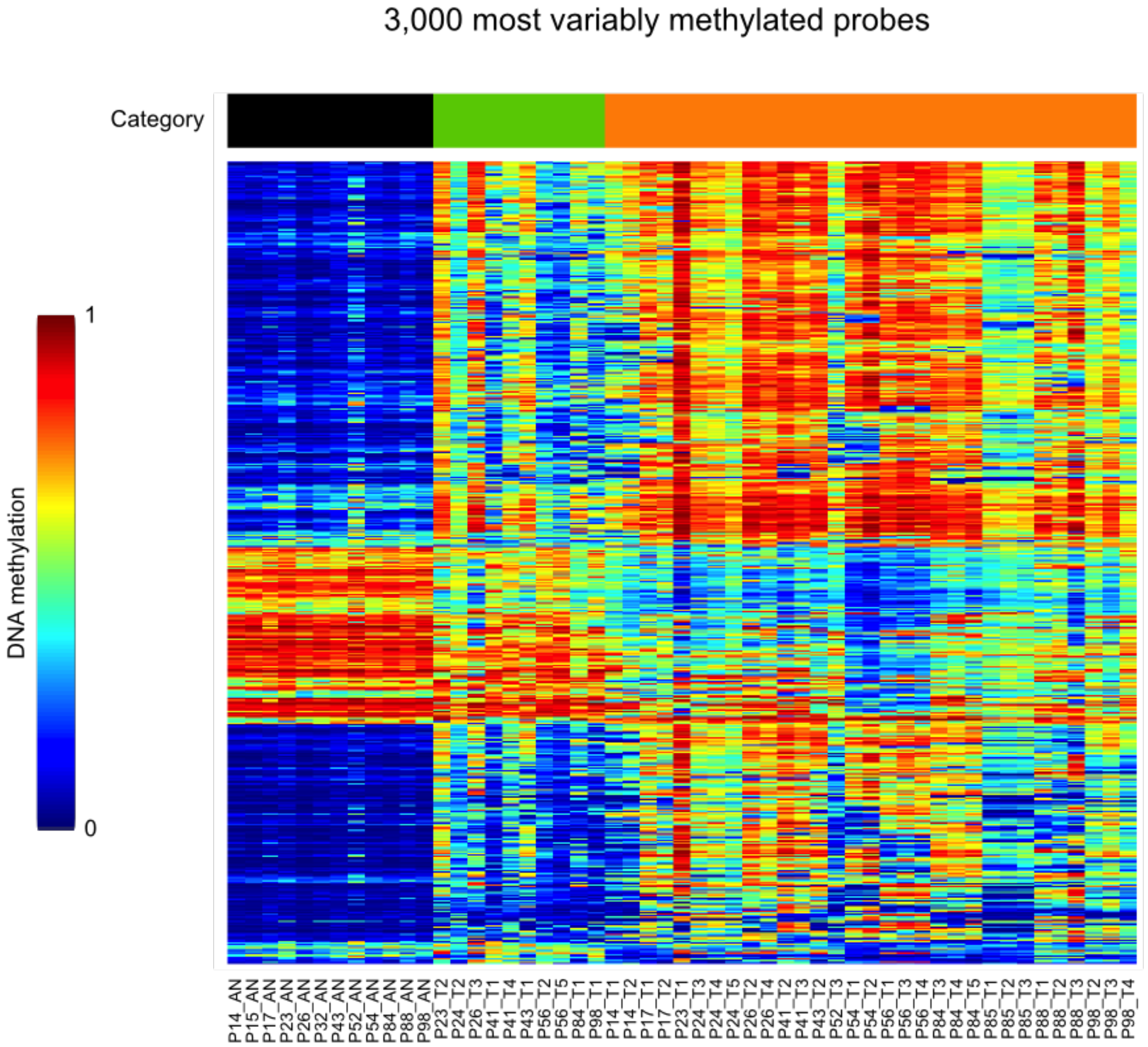
3,000 most variably methylated probes
Category
DNA methylation
1
0
P14_AN
P15_AN
P17_AN
P23_AN
P26_AN
P32_AN
P43_AN
P52_AN
P54_AN
P84_AN
P88_AN
P98_AN
P23_T2
P24_T2
P26_T3
P41_T1
P41_T4
P43_T1
P56_T2
P56_T5
P84_T1
P98_T1
P14_T1
P14_T2
P17_T1
P17_T2
P23_T1
P24_T3
P24_T4
P24_T5
P26_T2
P26_T4
P41_T2
P41_T3
P43_T2
P52_T3
P54_T1
P54_T2
P56_T1
P56_T3
P56_T4
P84_T3
P84_T4
P84_T5
P85_T1
P85_T2
P85_T3
P88_T1
P88_T2
P88_T3
P98_T2
P98_T3
P98_T4

**Figure S5**

**Gleason Score Distribution TCGA**

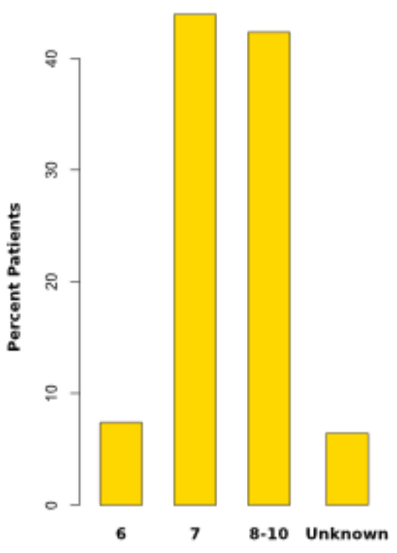


**Pathological T Stage Distribution TCGA**

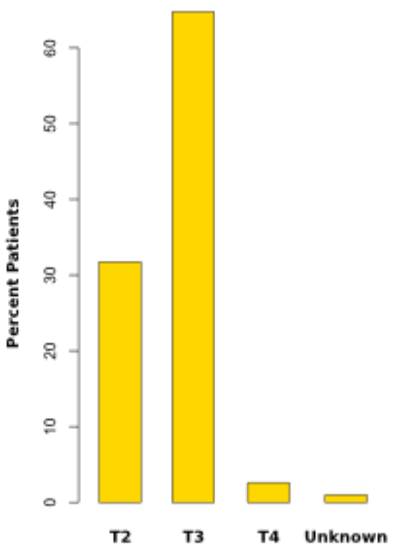

**Figure S6**

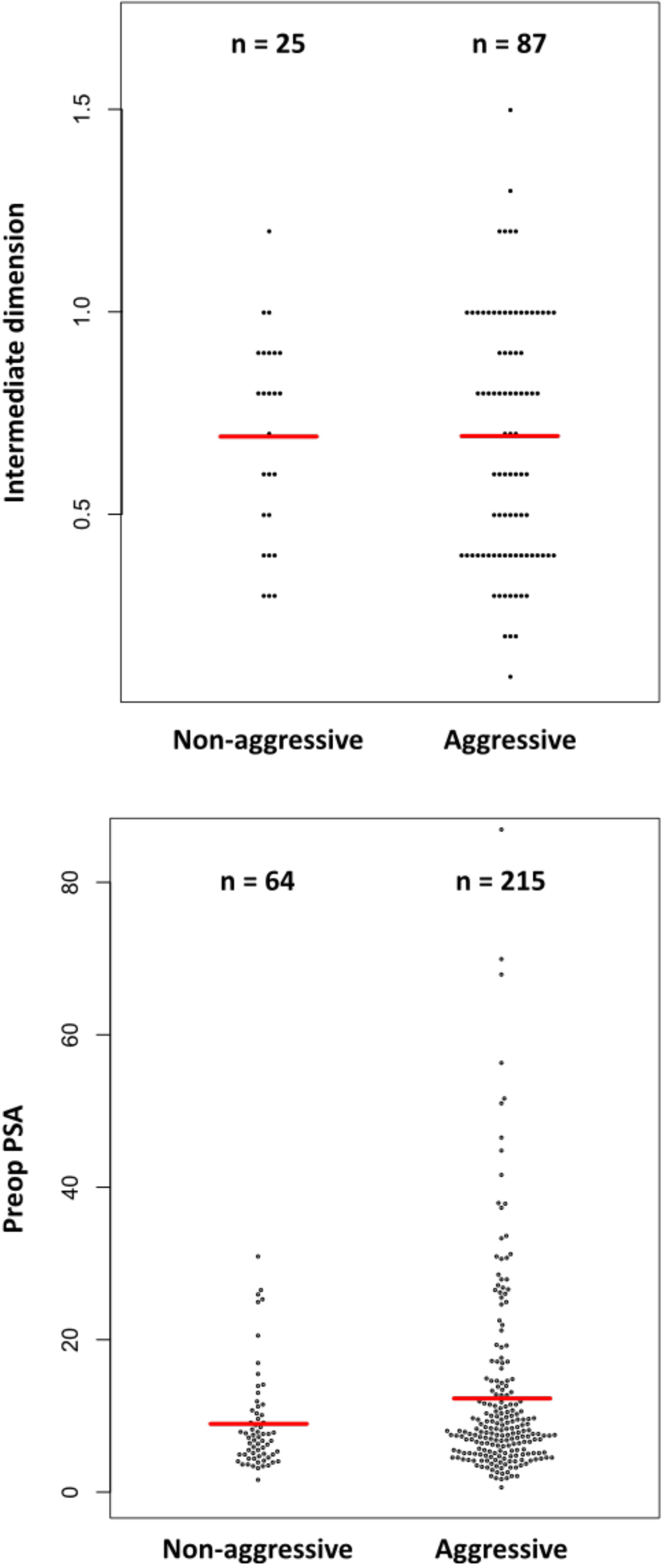
n = 25
n = 87
Intermediate dimension
0.5
1.0
1.5
Non-aggressive
Aggressive
n = 64
n = 215
Preop PSA
0
20
40
60
80
Non-aggressive
Aggressive

# Figure S7

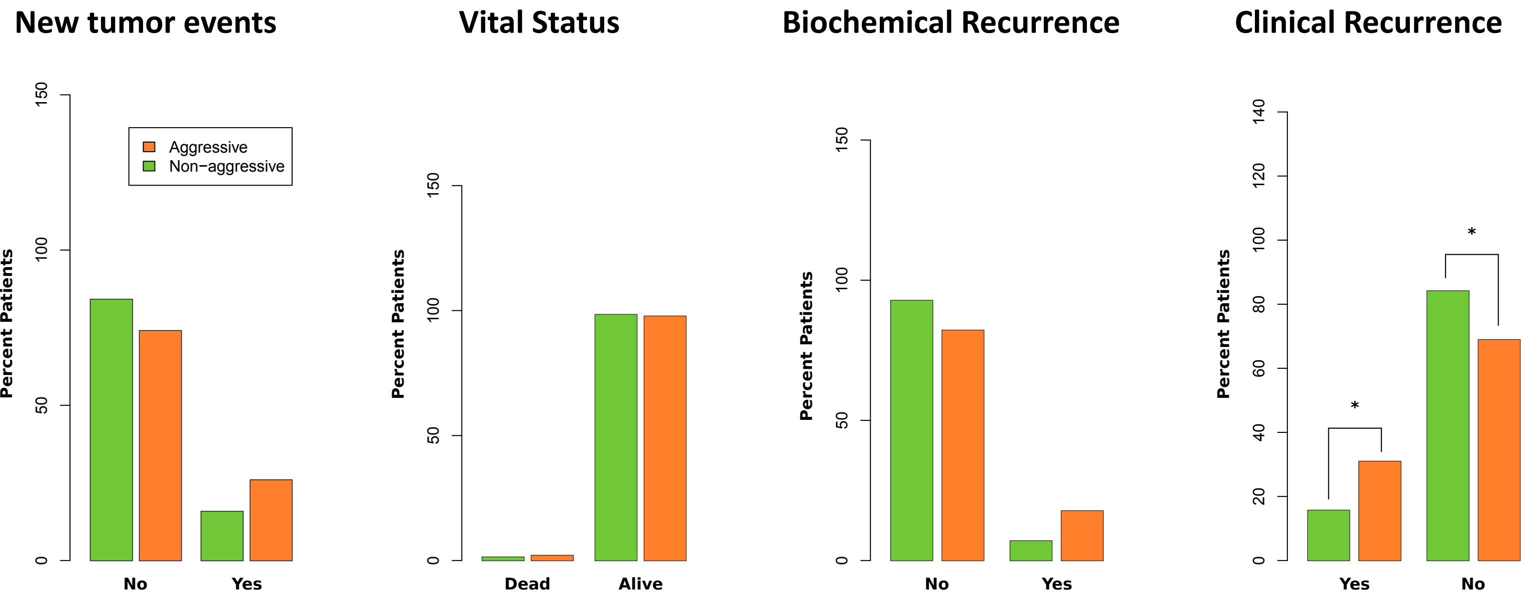


| New tumor events | No | (%) | Yes | (%) | Total |
|---|---|---|---|---|---|
| Aggressive | 110 | 74% | 39 | 26% | 149 |
| Non-aggressive | 37 | 84% | 7 | 16% | 44 |
| Total | 147 | | 46 | | 193 |
| | | | | | |
| **Vital status** | **Alive** | | **Dead** | | **Total** |
| Aggressive | 228 | 98% | 5 | 2% | 233 |
| Non-aggressive | 66 | 99% | 1 | 1% | 67 |
| Total | 294 | | 6 | | 300 |
| | | | | | |
| **Biochemical recurrence** | **No** | | **Yes** | | **Total** |
| Aggressive | 161 | 82% | 35 | 18% | 196 |
| Non-aggressive | 52 | 93% | 4 | 7% | 56 |
| Total | 213 | | 39 | | 252 |
| | | | | | |
| **Clinical recurrence** | **No** | | **Yes** | | **Total** |
| Aggressive | 129 | 69% | 57 | 31% | 186 |
| Non-aggressive | 48 | 84% | 9 | 16% | 57 |
| Total | 177 | | 66 | | 243 |

# Figure S8

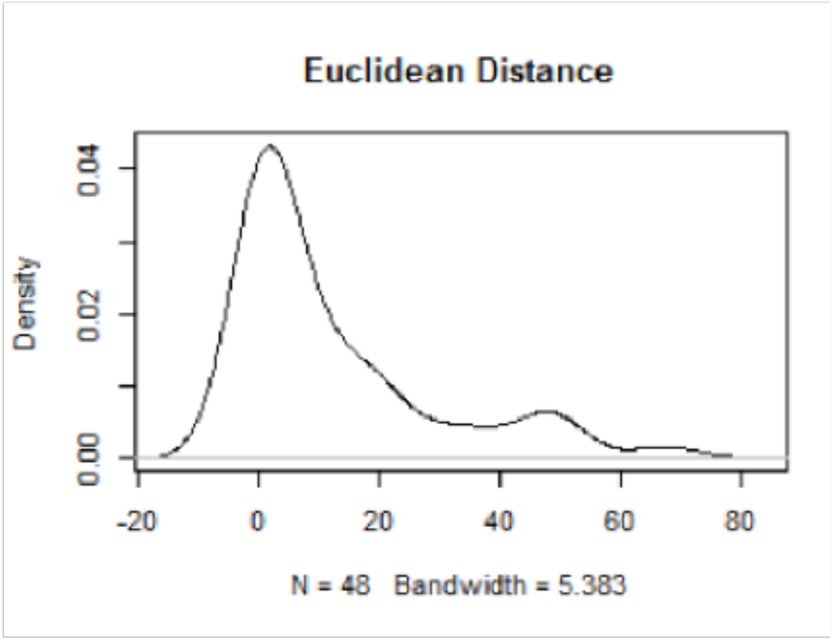
Euclidean Distance
Density
0.00
0.02
0.04
-20
0
20
40
60
80
N = 48 Bandwidth = 5.383

**Table S1. Patient Information.** LN, lymph node.

| Patient no. | Age (years) | Radical Prostatectomy (year) | Tumor Stage | Gleason Score | Nodal Stage | Malignant LNs | Patient Status (2015) |
|---|---|---|---|---|---|---|---|
| 14 | 64 | 2013 | pT3a | 4+5 | pN1 | 2/16 | Alive |
| 15 | 63 | 2007 | pT3a | 3+4 | pN1 | 2/9 | Alive |
| 17 | 61 | 2000 | pT3b | 3+5 | pN1 | 2/22 | Dead |
| 23 | 68 | 2004 | pT3 | 4+5 | pN1 | 14/49 | Dead |
| 24 | 66 | 2013 | pT3 | 3+4 | pN1 | 1/9 | Alive |
| 26 | 60 | 2013 | pT3b | 4+5 | pN1 | 3/45 | Alive |
| 32 | 66 | 2013 | pT3b | 4+4 | pN2 | 8/32 | Alive |
| 41 | 68 | 1999 | pT3b | 3+5 | pN1 | 6/11 | Dead |
| 43 | 62 | 2004 | pT3b | 5+4 | pN1 | 25/51 | Unknown |
| 52 | 75 | 2013 | pT3b | 5+4 | pN1 | 14/42 | Alive |
| 54 | 55 | 2008 | pT4 | 5+5 | pN1 | 4/8 | Dead |
| 56 | 57 | 2013 | pT3b | 5+4 | pN1 | 11/43 | Alive |
| 84 | 58 | 2008 | pT3b | 5+4 | pN1 | 4/50 | Alive |
| 85 | 61 | 2013 | pT3b | 4+4 | pN1 | 4/29 | Alive |
| 88 | 65 | 1991 | pT3b | 4+5 | pN2 | 5/20 | Dead |
| 98 | 79 | 2013 | pT3b | 4+5 | pN1 | 8/23 | Alive |

**Table S2. PC foci aggressiveness by patient.**

| Patient no. | Multiple subclones | No. of aggressive subclones | No. of non-aggressive subclones |
|---|---|---|---|
| 14 | No | 2 | 0 |
| 17 | No | 2 | 0 |
| 23 | Yes | 1 | 1 |
| 24 | Yes | 3 | 1 |
| 26 | Yes | 2 | 1 |
| 41 | Yes | 2 | 2 |
| 43 | Yes | 1 | 1 |
| 52 | ? | 1 | 0 |
| 54 | No | 2 | 0 |
| 56 | Yes | 3 | 2 |
| 84 | Yes | 3 | 1 |
| 85 | No | 3 | 0 |
| 88 | No | 3 | 0 |
| 98 | Yes | 3 | 1 |

**Table S3. 25-probe aggressiveness classifier.**

| Illumina CG ID | Specificity | Lambda coefficients | Methylation | UCSC REFGENE NAME | UCSC REFGENE GROUP | RELATION TO UCSC CPG ISLAND |
|---|---|---|---|---|---|---|
| cg00697992 | Normal | -1.614249 | Hypo | NXPH2 | Body | N_Shore |
| cg01272707 | Aggressive | 0.261565356 | Hyper | — | — | Island |
| cg01819167 | Aggressive | -0.21434696 | Hypo | — | — | — |
| cg01906055 | Aggressive | -6.01915727 | Hypo | NCAPH | TSS1500 | N_Shore |
| cg02160684 | Aggressive | -2.61406040 | Hypo | TRIB1 | Body | S_Shelf |
| cg02560085 | Non-aggressive | 0.05782731 | Hyper | PCDHA1-PCDHA8 | TSS200, Body | N_Shore |
| cg03195164 | Non-aggressive | 3.71373462 | Hyper | C3orf37 | Body | S_Shore |
| cg03456213 | Non-aggressive | 4.32173463 | Hyper | C9orf3 | Body | Island |
| cg04634417 | Aggressive | -0.81739612 | Hypo | PCDHA1-PCDHA8 | TSS200, Body | N_Shore |
| cg06024295 | Aggressive | 0.892107195 | Hyper | CPN1 | 1stExon, 5'UTR | — |
| cg11748187 | Non-aggressive | 0.35739768 | Hyper | TCF7L2 | Body | S_Shore |
| cg13300630 | Aggressive | 1.728928739 | Hyper | ROBO1 | Body | — |
| cg13944838 | Non-aggressive | -0.03066946 | Hypo | GFPT2 | Body | Island |
| cg14399930 | Aggressive | 1.911062008 | Hyper | FBXO47 | TSS200 | — |
| cg15132013 | Aggressive | -0.13732129 | Hypo | SKI | Body | S_Shelf |
| cg16469740 | Aggressive | 1.532165709 | Hyper | HDAC9 | Body | — |
| cg17004353 | Aggressive | -0.16606927 | Hypo | CARS | 5'UTR, Body | — |
| cg17032646 | Aggressive | 1.284803781 | Hyper | SLC6A17 | TSS1500 | N_Shore |
| cg18315943 | Aggressive | -0.00142294 | Hypo | — | — | Island |
| cg19550524 | Aggressive | -0.94604107 | Hypo | — | — | — |
| cg20399616 | Normal | -4.150844 | Hypo | BCAT1 | Body | Island |
| cg24517686 | Aggressive | -0.02269311 | Hypo | — | — | S_Shore |
| cg25961816 | Non-aggressive | -1.15467574 | Hypo | — | — | — |
| cg26447413 | Non-aggressive | 2.53402928 | Hyper | GAS1 | 1stExon | Island |
| cg27198013 | Non-aggressive | -0.48956758 | Hypo | RAI1 | 3'UTR | Island |
